# Effect of microstructure on the mechanical and damping behaviour of dragonfly wing veins

**DOI:** 10.1098/rsos.160006

**Published:** 2016-02-17

**Authors:** H. Rajabi, A. Shafiei, A. Darvizeh, J.-H. Dirks, E. Appel, S. N. Gorb

**Affiliations:** 1 Institute of Zoology, Functional Morphology and Biomechanics, Christian-Albrechts-University, Kiel, Germany; 2 Department of Mechanical Engineering, The University of Guilan, Rasht, Iran; 3 Young Researchers and Elite Club, Lahijan Branch, Islamic Azad University, Lahijan, Iran; 4 Department of New Materials and Biosystems, Max Planck Institute for Intelligent Systems, Stuttgart, Germany; 5 Department for Biomimetics, Bremen University of Applied Sciences, Bremen, Germany

**Keywords:** insect wing, vein, resilin, cuticle, finite-element modelling, damping, rigidity

## Abstract

Insect wing veins are biological composites of chitin and protein arranged in a complex lamellar configuration. Although these hierarchical structures are found in many ‘venous wings' of insects, very little is known about their physical and mechanical characteristics. For the first time, we carried out a systematic comparative study to gain a better understanding of the influence of microstructure on the mechanical characteristics and damping behaviour of the veins. Morphological data have been used to develop a series of three-dimensional numerical models with different material properties and geometries. Finite-element analysis has been employed to simulate the mechanical response of the models under different loading conditions. The modelling strategy used in this study enabled us to determine the effects selectively induced by resilin, friction between layers, shape of the cross section, material composition and layered structure on the stiffness and damping characteristics of wing veins. Numerical simulations suggest that although the presence of the resilin-dominated endocuticle layer results in a much higher flexibility of wing veins, the dumbbell-shaped cross section increases their bending rigidity. Our study further shows that the rubber-like cuticle, friction between layers and material gradient-based design contribute to the higher damping capacity of veins. The results of this study can serve as a reference for the design of novel bioinspired composite structures.

## 1. Introduction

Dragonfly wings are fascinating biological composite structures consisting of an ultra-thin membrane reinforced with a network of hollow veins. These mostly tubular veins divide the wing into several small parts called cells. The veins have different sizes and arrangements and can be classified into three main groups: (i) thick longitudinal veins that extend from wing base to wing tip, (ii) thin and short cross-veins which interconnect the adjacent longitudinal veins, and (iii) marginal ambient veins.

The veins found in insect wings combine many different biomechanical functions within one single structure. One of the main functions of the veins is obviously to provide the structural framework of the wing [1,2]. Indeed, they are highly specialized elements which enable insects to withstand large aerodynamic forces in flight [3,4].

Previous studies suggested that cross-veins in insect wings act as stiffening elements providing lateral support for longitudinal veins [5]. Cross-veins can also provide crack arresting mechanisms by temporarily stopping the propagation of induced cracks, and therefore they increase the wing's structural fracture toughness [6,7].

It has been shown that longitudinal veins, especially those situated close to the leading edge, dramatically enhance the stiffness of wings in the spanwise direction [2,5,8]. Numerical simulations indicate that the presence of longitudinal veins also improves the natural frequencies of both fore- and hindwings of dragonfly *Orthetrum Sabina* (Anisoptera, Libellulidae) approximately twofold, and therefore avoids structural failure due to resonance [9]. What are the underlying factors that cause the unique mechanical behaviour exhibited by insect wing veins?

The microstructure of dragonfly wing veins has been rarely reported in the literature [10–14]. Previous microscopic examinations indicated that veins in dragonfly wings possess a sandwich microstructure that mainly consists of two chitinous shells and one fibrous protein layer [13,14]. Later, it was shown that these three layers contain a hierarchical microstructure which consists of several concentric chitinous shells and clusters of protein fibrils oriented in the circumferential direction [11]. However, recent morphological investigations using a combination of scanning electron microscopy (SEM), transmission electron microscopy (TEM) and fluorescence light microscopy (FLM) have demonstrated that vein microstructure is much more complex [10]. Veins have a lamellar organization and consist of fibrous composite materials of chitin supplemented by proteins, including resilin.

### 1.1. Microstructure of the vein

**Figure 1.**
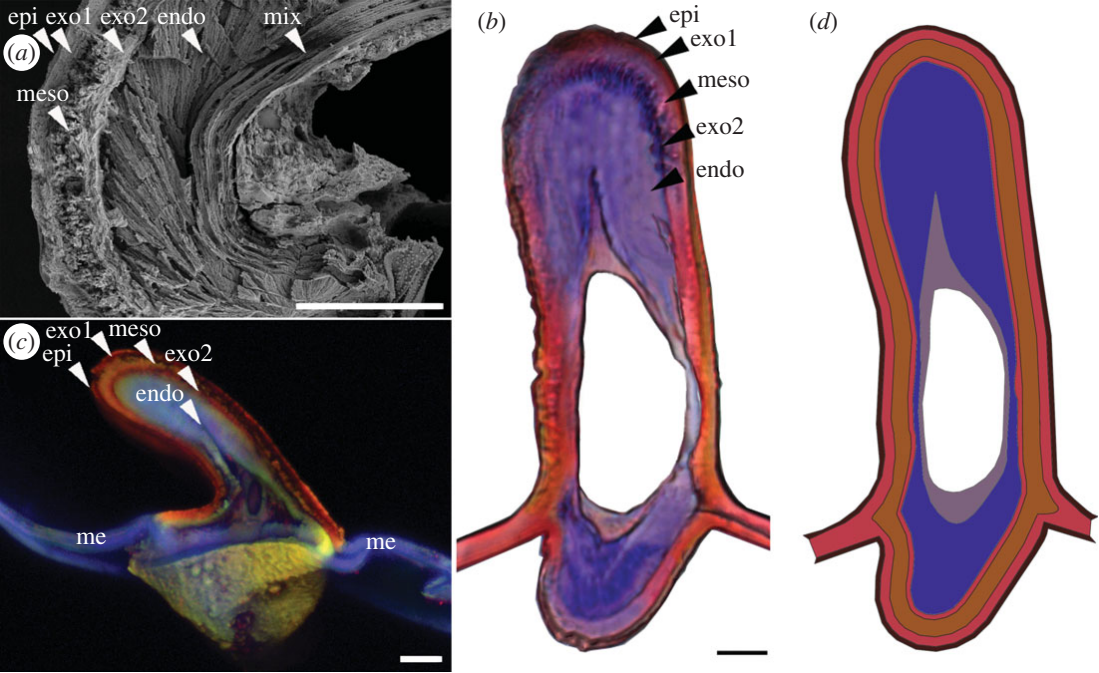
(*a*) SEM, (*b*) bright-field light microscopy and (*c*) confocal laser scanning microscopy (CLSM) images of the cross sections of the wing vein [10] of the dragonfly *Sympetrum vulgatum* (Anisoptera, Libellulidae). The blue and red colours represent the endocuticle and exocuticle layers of the vein. (*d*) Developed finite-element model of the vein based on microscopic observations. epi, epicuticle; exo1, outer exocuticle; meso, mesocuticle; exo2, inner exocuticle; endo, endocuticle; mix, mixed layer; me, membrane. Scale bars (*a*–*d*), 10 µm.

Microscopic studies on *Sympetrum vulgatum* (Anisoptera, Libellulidae) and *Matrona basilaris basilaris* (Zygoptera, Calopterygidae) revealed that dragonfly wing veins may have up to six distinguishable layers [10]: (i) an epicuticle layer (including the waxy layer) that has a thickness up to 0.8 µm; (ii) a highly sclerotized exocuticle layer with a maximum thickness of 3.9 µm; (iii) a less sclerotized intermediate layer (mesocuticle) with a thickness up to 6.9 µm; (iv) a less dense and thinner, up to1.4 µm, exocuticle layer; (v) a relatively thick, up to 16.4 µm, endocuticle layer; and (vi) a combination of both exo- and endocuticle in a serial arranged layered structure, called the mixed layer (up to 4 µm). There is also a layer of epidermal cells in the interior surface of the hollow microstructure of the vein. An SEM image showing the mentioned cuticular layers in the vein microstructure is presented in figure 1*a*. A bright-field light microscopy image is given in figure 1*b*. In this figure, the vein section is stained with Azan staining, which differentiates between sclerotized (red) and less sclerotized cuticle (blue). Strongly sclerotized cuticle is refractory to the colour (e.g. exocuticle). Confocal laser scanning microscopy confirmed the presence of resilin, a rubber-like protein in the endocuticle layer (figure 1*c*). The blue colour of the endocuticle, in figure 1*c*, is due to the blue autofluorescence characteristic of resilin under ultraviolet (UV) light excitation [15,16]. Although one might suppose that resilin increases the flexibility of the vein structure, the precise functional role of this rubber-like material as well as some other features, such as frictional properties at the interface between the layers, the dumbbell-shaped cross section, exact material composition and specific layered structure, are still unknown.

This article is the first attempt at a quantitative numerical investigation of the effects of these variables on the mechanical and damping behaviour of insect wing veins. For this purpose, the data from microscopic observations were used to develop and analyse a series of three-dimensional finite-element (FE) models of veins with different structural features. The results suggest that applying the same design concepts found in biological systems may improve the current design strategies of technical composite structures.

## 2. Material and methods

### 2.1. Finite-element modelling of different wing models

ABAQUS/Standard v. 6.1 was used to develop eight FE models of the libellulid dragonfly *S. vulgatum* wing veins. These numerical three-dimensional models simulate a diversity of microstructural features of the veins.

Model 1 is the most comprehensive one, containing the highest number of features of a real vein including its layered structure and material compositions. This model, which is shown in figure 1*d*, was developed based on detailed measurements on the microscopic images of the vein cross section shown in figure 1. The six cuticular layers, mentioned in §1.1, are included in the model and a strong interaction is defined between them. Model 1 serves as the reference model in this study.

In model 2, the resilin-dominated endocuticle layer from model 1 was replaced by an exocuticle layer. Comparison of the results from the numerical analysis of this model with those of model 1 can address the effect of the rubber-like materials in the deeper layers of the vein.

In order to study the mechanical effect of contact between the internal layers of the vein, we developed model 3 with a frictionless contact between the layers. This type of contact allows the sliding of the layers on each other.

A circular and an elliptical vein model (models 4 and 5) were developed to estimate the influence of the dumbbell-shaped cross section on the mechanical behaviour of the vein. The cross-sectional area of these two models was considered to be equal to that of model 1. The elliptical vein model (model 5) has the same ratio of maximum to minimum diameter as model 1.

Models 6 and 7 are fully made of endocuticle and exocuticle, respectively, to determine the effect of combination of these cuticular layers in the vein structure.

The influence of the layered structure on the mechanical behaviour of the vein is evaluated through the comparison between the results obtained from the processing of model 1 with those of model 8. The latter is made of an equivalent homogeneous material, which is calculated using the method proposed by Beer *et al.* [17]. The characteristics of the models together with their two-dimensional cross-sectional views are listed in table 1. The input files of the FE models, used in this study, are available in the electronic supplementary material (model S1–S4).

**Table 1.** Characteristics of the models. The symbols ✓ and × indicate the presence and absence of the mentioned feature, respectively. The intensity of the grey colour in the schematic views shows a higher stiffness.

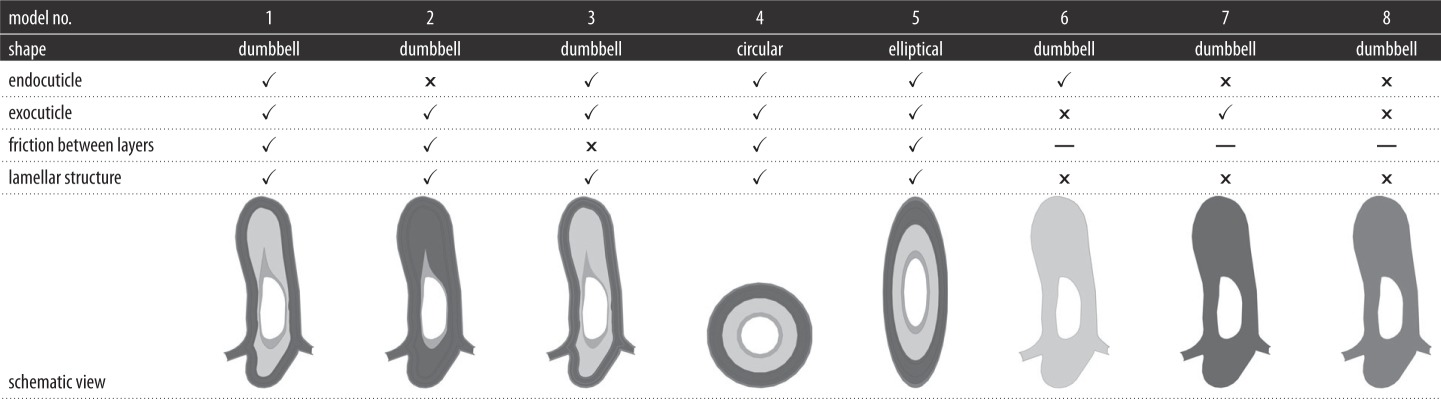

Three-dimensional hexahedral continuum elements with first-order interpolation method, which are recommended element types to study the stress and deformation fields, are used for FE modelling [18]. A reduced integration scheme is employed to decrease the computational cost. Furthermore, we use an enhanced hourglass control to prevent the possible mesh instability due to the use of reduced integration elements.

### 2.2. Material properties

There are only very limited experimental data available in the literature on the mechanical properties of insect wings and particularly wing veins [1,6,19,20]. Therefore, in this study, we used the material properties that are obtained from different experiments and for different insect species. Although the selected properties may not exactly match the properties of dragonfly wing vein materials, our previous numerical studies showed that even applying basic biomechanical properties in an accurate geometrical model can suffice to simulate the mechanical behaviour of biological structures [7,21–26]. That is why, for an exploratory comparative study, such as the one presented here, it is not absolutely necessary to use the exact values of the material properties.

Previous experiments showed that, before failure, the deformation of insect wings typically occurs in an elastic manner [1,6,12,27,28]. Therefore, a linear elastic material model was used here to simulate the mechanical behaviour of the materials forming the insect wing veins.

Based on the data obtained from nanoindentation testing, the epicuticle layer was considered to have an elastic modulus of 2.85 GPa [29]. A Young's modulus of 6.17 GPa was assigned to the exocuticle layer [19]. This value corresponds to the stiffness of desiccated tibia cuticle of the locust *Schistocerca gregaria*. Young's modulus of endocuticle was taken as 2 MPa [30]. We chose an average stiffness of 3 GPa for the mixed layer. The same density, 1200 kg m^−3^, was used for both epicuticle and exocuticle layers [20]. However, the endocuticle was assumed to have a lower density of 1000 kg m^−3^ (equal to the density of water) [19]. Poisson's ratio of all layers was taken as 0.3 [31].

The damping properties of insect exo- and endocuticle have been taken from the mechanical tests performed by Dirks & Dürr [32]. Based on the experimental results, we considered a damping ratio of 0.02 and 1 for the exocuticle and endocuticle, respectively. The same damping properties as those of the exocuticle were assigned to the epicuticle layer. It is also important to note that the equivalent material and damping properties of model 8 were determined according to the equivalent stiffness and damping method [33].

### 2.3. Loading and boundary conditions

In our study, loading conditions were selected to induce deformations smaller than 5% of the length of the models, as expected in real flight [34]. Veins were modelled as cantilever beams with the same length (1 mm). A 0.25 × 10^−6^ m displacement boundary condition was employed to produce a tensile stress in the axial direction. Bending was accomplished by applying a displacement boundary condition of 0.3 × 10^−6^ m at the free end of the models and parallel to the cross section. We used a rotation boundary condition (0.75°) to exert a torsional moment about the longitudinal axis of the veins. These three loading scenarios were taken into account to measure the axial, bending and torsional rigidities of our vein models. Although these loading conditions are typically different from those applying to insect wings during flight, they give us a useful tool to simulate the overall normal and shear mechanical stresses experienced by veins in flight. These loading scenarios also enabled us to comparatively study the mechanical performance of the vein models under different conditions.

For dynamic analysis, the loading and boundary conditions were chosen to simulate the experiment conducted by Dirks & Dürr [32] on the stick insect antenna. The vein models with an identical length (2 mm) were fixed on one side and an initial deflection was imposed on their other side. The return movement of the samples to their original position after the induced bending was numerically predicted.

We used a static-general and a modal dynamic analysis type to simulate the static and dynamic behaviour of the models, respectively. In all analyses, the effect of possible geometric nonlinearity was taken into account. However, owing to the relatively small deformations applied to the models, no considerable difference was observed between the results from the linear and nonlinear analyses.

### 2.4. Damping model

In this study, Rayleigh damping method was employed to define the damping behaviour of the vein models using a modal dynamic analysis. Rayleigh damping that is usually used for proportional damping systems linearly connects the damping matrix to the mass and stiffness matrices [35]. This pre-assumption together with the orthogonality condition of the vibration modes results in the following relation between the damping ratio (*ζ_i_*) and the natural frequencies (*ω_i_*) of an undamped system [18]:

2.1 $$\zeta_{i}=\frac{\alpha }{2\omega_{i}}+\frac{\beta \omega_{i}}{2},$$

where *α* and *β* are the mass proportional and stiffness proportional Rayleigh damping coefficients, respectively. Considering *ω*_1_ and *ω_m_* as the first and *m*th natural frequency of the system and assigning an equal damping ratio (*ζ* = *ζ*_1_ = *ζ_m_*) for both natural frequencies gives

2.2 $$\alpha =2\zeta \frac{\omega_{1}\omega_{m}}{\omega_{1}+\omega_{m}}$$

and

2.3 $$\beta =2\zeta \frac{1}{\omega_{1}+\omega_{m}}.$$

Using the above equations, the Rayleigh coefficients are determined for the exo- and endocuticle layers. In order to improve the accuracy of the predictions, the obtained values were varied and the results were compared with the previously available experimental data [32]. The Rayleigh coefficients obtained for different layers in the vein models are presented in the electronic supplementary material, table S1.

## 3. Results

**Figure 2.**
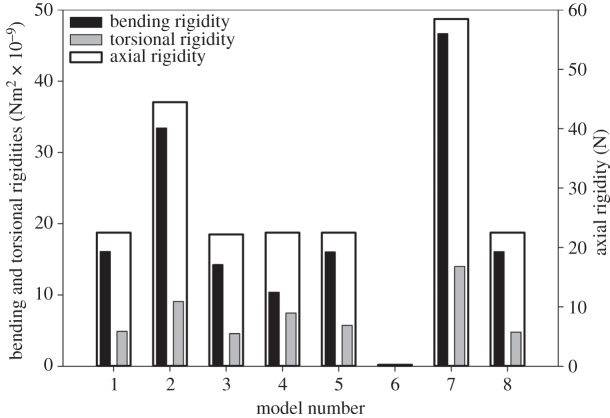
Axial, bending and torsional rigidities of the vein models.

Comparison of the axial, bending and torsional rigidities obtained from the FE analysis of the developed models is presented in figure 2. As shown in this figure, replacement of the endocuticle layer by a layer of exocuticle results in an increase in the measured rigidities by approximately two times. Based on the results, the interaction between the layers has only a small influence on the rigidity of the vein structure. Indeed, removal of the strong contact between the layers in the vein microstructure, in model 3, induces a negligible decrease in the measured rigidities.

The axial rigidity is not changed by transforming the shape of the vein cross section to a circle with the same area (model 4). However, this transformation is associated with an increase in the torsional rigidity and a decrease in the bending rigidity. Changing the shape of the vein cross section to an ellipse (model 5), described in §2.1, does not affect the vein rigidities.

As expected, in comparison with model 1, models 6 and 7, which are made of only endocuticle and exocuticle, respectively, exhibit very different rigidities. Model 6 (endocuticle model) shows axial, bending and torsional rigidities that are much smaller than the realistic vein model (model 1). However, model 7 (the exocuticle model) has rigidities that are at least 2.6 times higher than those of model 1. The model with equivalent material properties and without material gradient (model 8) exhibits the same mechanical behaviour as model 1.

Table 2 summarizes the changes in the vein rigidities due to the physical and geometrical changes applied in our numerical models. As previously mentioned, all changes in this table are described in comparison to the realistic vein model (model 1). It is also important to note that the comparison of the results indicated a good agreement between simulations and the data from previous experiments on dragonfly wing veins [36].

**Table 2.** Changes in the axial, bending and torsional rigidities of the vein models in comparison to model 1.

model no.	induced change	change in the axial rigidity (in times)	change in the bending rigidity (in times)	change in the torsional rigidity (in times)
2	replacing an exocuticle layer instead of endocuticle layer	1.98	2.08	1.86
3	removing friction between the internal layers in the vein microstructure	0.99	0.89	0.94
4	transforming the dumbbell-shaped cross section to a circular cross section	—	0.65	1.53
5	transforming the dumbbell-shaped cross section to an elliptical cross section	—	0.99	1.17
6	replacing all vein layers with only one endocuticle layer (uniform material distribution)	0.0004	0.0004	0.0004
7	substituting all vein layers with only one exocuticle layer (uniform material distribution)	2.60	2.90	2.86
8	using an average material property with a uniform distribution instead of a layered structure	—	—	—

**Figure 3.**
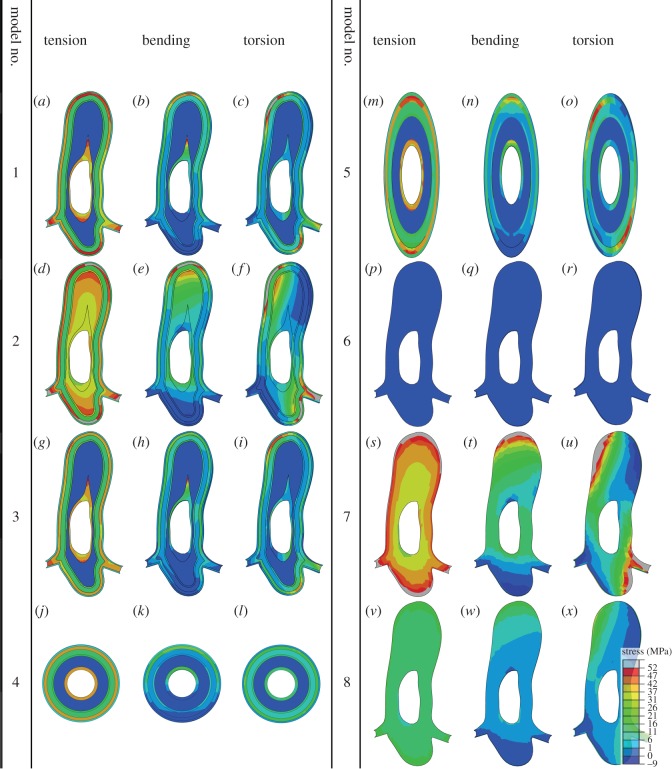
Distribution of the maximum principal stress at the fixed end of the vein models under (*a*,*d*,*g*,*j*,*m*,*p*,*s*,*v*) tension, (*b*,*e*,*h*,*k*,*n*,*q*,*t*, *w*) bending and (*c*,*f*,*i*,*l*,*o*,*r*,*u*,*x*) torsion.

The distribution of the maximum principal stress in the cross section of the models, at the fixed end, under the same tensile, bending and torsional deformations is presented in figure 3. It is demonstrated that in all loading conditions, model 2 (model with no endocuticle layer) experiences considerably higher levels of stress in comparison with model 1 (comprehensive model). The latter also predicts high local stress concentrations that usually occur in the exocuticle layer. The stress distribution in model 3 (model with weak interaction between layers) is not qualitatively different from that of the comprehensive vein model, but the stress level in this model is comparatively lower.

In contrast to model 1 (comprehensive model), a more uniform stress distribution is observed in model 4 (circular model). Especially under torsion, the stress is evenly distributed within each layer of the circular model. The stress pattern in model 5 (elliptical model) is almost similar to that obtained from the comprehensive vein model (model 1). In all loading conditions, model 6 (fully endocuticle model) shows considerably lower stress level and model 7 (fully exocuticle model) exhibits a remarkably higher stress level in comparison to the realistic vein model (model 1). Interestingly, more uniform stress patterns than those of model 1 are found in the model with equivalent material properties (model 8), under axial, bending and torsional deformations.

**Figure 4.**
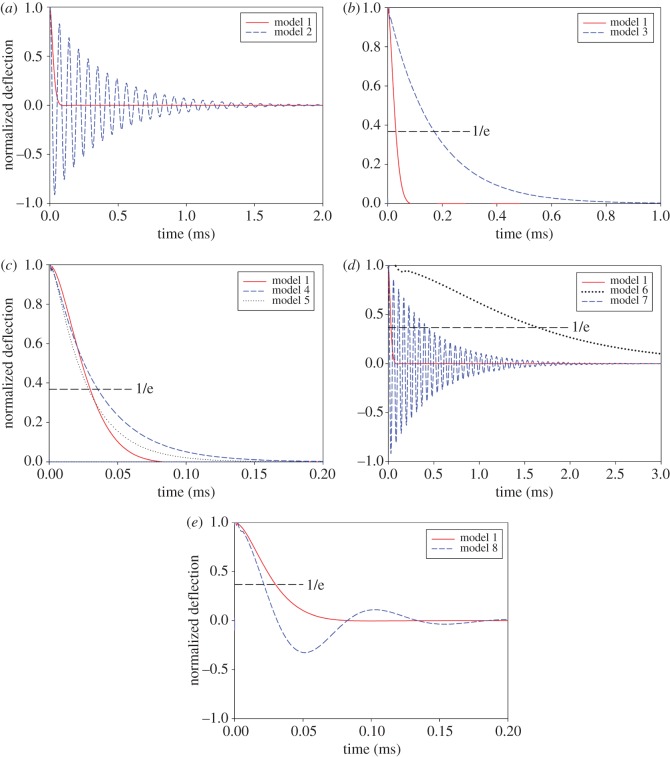
Decay time histories of the vein models. Damping behaviour of models (*a*) 2, (*b*) 3, (*c*) 4 and 5, (*d*) 6 and 7, and (*e*) 8 in comparison to model 1. Models containing no endocuticle (*a,d*) and model 8 with equivalent material properties (*e*) show an under-damped behaviour. Models containing endocuticle (*a–d*) reveal an over-damped dynamic response.

Figure 4 presents the typical decay time histories obtained from the dynamic analysis of the vein models. The results from the comprehensive vein model (model 1) indicate that the vein structure is an over-damped system. This finding is in accordance with the observations of Dirks & Dürr [32] on the stick insect antenna. Considering the relatively similar cuticular microstructure of antennae and wing veins [10,32,37], the quantitatively same dynamic behaviour of both these structures seems to be a reasonable observation.

Comparison of the damping characteristics of model 1 (comprehensive model) and model 2 (model with no endocuticle layer) is presented in figure 4*a*. This figure shows how the removal of the endocuticle layer causes a transition from an over-damped to an under-damped state. Decreasing the friction between layers in the vein microstructure (model 3) results in a small increase in the effective damping ratio of the vein system (figure 4*b*). Here, in order to perform quantitative analysis of the exponential decays, we use the decay time constant, which is the time required for a system to return to 36.8% of its initial deformation. The time constants for the comprehensive vein model (model 1) and the model with the weak contact between layers (model 3) are 0.03 ms and 0.17 ms, respectively.

Based on the numerical results, changing the shape of the cross section does not significantly change the damping characteristics of the vein (figure 4*c*). However, the difference between the time constants of the comprehensive vein model (model 1) and the elliptical model (model 5) is much smaller than that of the comprehensive vein model (model 1) and the circular one (model 4).

As illustrated in figure 4*d*, in contrast to model 1, model 6 that is fully made of endocuticle has an extremely slow decay. This model has a decay time constant of 1.7 ms which is considerably larger than that of the comprehensive model (0.03 ms). In contrast to these two models, the fully exocuticle model (model 7) is an under-damped system exhibiting strong oscillations. Interestingly, the use of equivalent material and damping properties in model 8 causes a transition to an under-damped regime with a few oscillations (figure 4*e*).

## 4. Discussion

### 4.1. Effect of the resilin-dominated endocuticle layer

The significant increase in the axial, bending and torsional rigidities of the vein after replacement of the endocuticle layer by an exocuticle layer, in model 2, was expected due to the higher stiffness of exocuticle. This finding, together with the higher levels of stress in model 2 compared with the comprehensive vein model (model 1), suggests two possible roles for the resilin-dominated endocuticle: (i) enhancing the flexibility of the vein structure, by approximately two times, and (ii) preventing stress concentrations. The latter has an important effect on improving the load-bearing ability of the vein under large deformations. Based on the fact that desiccation makes the soft endocuticle similar to the stiff exocuticle [30,32], the replacement of the endocuticle layer by the exocuticle one, in model 2, further allows us to estimate the possible mechanical effects caused by desiccation.

Comparison of the damping behaviour of model 1 with that of model 2 showed how the presence of resilin in the vein structure leads to an over-damped non-oscillatory behaviour (figure 4*a*). This means that in a normal condition, after an initial mechanical deflection, the vein returns to its rest position without or with only low overshooting. However, after desiccation, the vein exhibits a damped harmonic oscillating motion. Given the negative influence of mechanical oscillations on the stability of a dynamic system, this finding suggests another essential role for resilin in insect wings, except enhancing the flexibility, to improve the damping properties of the wing structure. This may lead to an improved dynamic performance and consequently higher fatigue resistance.

### 4.2. Effect of friction between layers

The mechanics of the contact between the layers in the vein microstructure is still unknown. However, microscopic images did not show visible structural discontinuities between the layers in the vein microstructure, which suggests relatively strong interactions between them. The small reduction in the stiffness of the vein by removing the friction between layers (model 3) is due to the sliding of these layers on each other (figure 2). In other words, in this case, no force is required to overcome the friction and this increases the deformability of the vein structure. This phenomenon also results in a slightly lower stress level in model 3 (figure 3*g*–*i*).

On the other hand, the results from the dynamic analysis of model 3 showed that removing the friction between layers increases the time constant of the vein over-damped system, and consequently the model is damped at a later point of time (figure 4*b*). In other words, the results suggest that one of the advantages of the assumed strong interaction between the layers is that the vein system is damped in a quite short time. The shorter decay time of the vein results in the faster stability of the entire wing.

### 4.3. Effect of geometry

Longitudinal and cross-veins in different parts of dragonfly wings have different cross sections [2,12,26,38]. However, longitudinal veins with dumbbell-shaped cross sections are very common, not only in dragonflies, but also in other insects [21,39].

The axial rigidity of a structure depends on the cross-sectional area and the stiffness of the material from which it is made. Therefore, considering the same cross-sectional area and similar material composition of the dumbbell-shaped, circular and elliptical models, the axial rigidity is not influenced by the shape of the vein cross section. However, comparison of the results from the comprehensive vein model (model 1) with those of circular and elliptical models (models 4 and 5), in figure 2, suggests that the dumbbell-shaped cross section of the vein is a morphological adaptation to improve the bending rigidity of the wing, although this shape of the cross section leads to a small decrease in the torsional rigidity. The increased bending rigidity is due to the higher second moment of area of the vein with the dumbbell-shaped cross section compared with the circular and elliptical veins for a given volume.

### 4.4. Effect of material gradient and layered structure

Comparison of the rigidity of the comprehensive vein model (model 1) with those of the endocuticle and exocuticle models (models 6 and 7) indicates that the combination of endo- and exocuticle layers in the vein structure provides a balance between flexibility and stiffness (figure 2). Considering the role of flexibility in enhancing the aerodynamic performance of insect wings [40], a stiff wing containing no endocuticle would not be able to produce an optimum lift-to-drag ratio. On the other hand, the veins made of only soft endocuticle cannot resist the external forces, and therefore do not give the wings the desired functionality. Investigation of the stress distribution in these three models also suggests that the presence of the resilin-dominated endocuticle layer reduces the stress level within the vein, and therefore enables it to withstand larger deformations.

The distribution of the stress in the real vein model (model 1) indicated that, in all loading conditions, the maximum stress is mainly maintained by the outer stiff layer (figure 3). Therefore, the presence of the layered structure in the real vein can be explained by specific developmental reasons for insect cuticular structures in general or/and by the need to have a stiff cover that is capable of resisting contact damages. On the other hand, the soft internal layer of the vein enhances the flexibility of the whole vein structure. Furthermore, it provides higher toughness and acts as a barrier to the propagation of cracks formed in the outer stiff layer [36].

The results from the dynamic analysis showed that the combination of the non-oscillatory endocuticle (model 6) with the under-damped harmonic oscillatory exocuticle (model 7) results in a mechanical system (model 1) that is very close to the critical damping condition. This result has a great importance, since it shows that a vein model containing both exo- and endocuticle has a lower decay time constant between all the developed models.

Besides the material composition, the layered structure seems to benefit the vein by improving the damping characteristics. Interestingly, using equivalent damping properties in model 8 does not produce the same dynamic behaviour as the model with heterogeneous damping properties of layers (model 1). Indeed, a uniform distribution of equivalent material and damping properties in the vein structure causes a relatively weaker damping effect (figure 4*e*). This difference in the dynamic behaviour of models 1 and 8, which is due to their different natural frequencies, leads us to suggest a possible role for the layered structure of the vein as a mechanism to enhance the damping performance of the vein system.

## 5. Conclusion

This article presents the first numerical simulation of the mechanical and damping behaviour of dragonfly wing veins, which represent the biological composite structure with a lamellar organization. The findings demonstrate the effects of some microstructural features on the mechanical response of the veins which would be difficult to quantify experimentally. Using a series of FE models, we showed that the friction between layers in the vein microstructure and the layered structure of wing veins result in better damping characteristics. Furthermore, it was found that desiccation dramatically reduces the flexibility of the veins and increases their decay time constant. The numerical results also suggest that the combination of the endo- and exocuticle in real veins may provide a relatively uniform stress distribution, an improved damping performance and a balance between flexibility and stiffness.

Our results show that in order to produce a fibre-reinforced composite structure, using fibres with a layered structure may lead to improved damping properties. However, due to lower stress concentration, a composite structure containing fibres with a uniform material distribution exhibits a higher load-bearing capacity. Future numerical simulations are planned to clarify the effect of the layered structure on the fracture behaviour of insect wing veins.

## Supplementary Material

Table S1: Rayleigh damping coefficients and natural frequencies calculated for different layers in vein models.

## Supplementary Material

Model S1: The input file of the FE models 1-3.

## Supplementary Material

Model S2: The input file of the FE model 4.

## Supplementary Material

Model S3: The input file of the FE model 5.

## Supplementary Material

Model S4: The input file of the FE model 6-8.

## Data accessibility

All supporting data are made available either in the manuscript or the electronic supplementary material.
