# Supplementary material for: Effect of microstructure on the mechanical and damping behaviour of dragonfly wing veins
Source: R Soc Open Sci. 2016 Feb 17;3(2):160006. doi: 10.1098/rsos.160006 (PMC4785991; doi:10.1098/rsos.160006)
Supplement: Table S1: Rayleigh damping coefficients and natural frequencies calculated for different layers in vein models. [file rsos160006supp1.docx]

Suppl. Table 1. Rayleigh damping coefficients and natural frequencies calculated for different layers in vein models.

| Model no. | Layer no. | α | β | Natural frequency (rad/s) |
| --- | --- | --- | --- | --- |
| 1, 3 | 1 | 282.7 | 1.3×10^-6^ | 11309 |
|  | 2 | 761 | 5×10^-7^ | 31095 |
|  | 3 | 456 | 8.7×10^-7^ | 20527 |
|  | 4 | 568.9 | 6.7×10^-7^ | 23279 |
|  | 5 | 1054 | 8×10^-4^ | 760 |
|  | 6 | 1373 | 2.5×10^-7^ | 50749 |
| 2 | 1 | 282.7 | 1.3×10^-6^ | 11309 |
|  | 2 | 761 | 5×10^-7^ | 31095 |
|  | 3 | 456 | 8.7×10^-7^ | 20527 |
|  | 4 | 568.9 | 6.7×10^-7^ | 23279 |
|  | 5 | 1837 | 1.8×10^-7^ | 66124 |
|  | 6 | 1373 | 2.5×10^-7^ | 50749 |
| 4 | 1 | 794 | 5×10^-7^ | 38264 |
|  | 2 | 1704 | 2.3×10^-7^ | 83145 |
|  | 3 | 2750 | 1.3×10^-7^ | 107002 |
|  | 4 | 2009 | 1.8×10^-7^ | 79092 |
|  | 5 | 2604 | 2.5×10^-4^ | 1646 |
|  | 6 | 2254 | 1.7×10^-7^ | 98696 |
| 5 | 1 | 621 | 6.4×10^-7^ | 30347 |
|  | 2 | 1732 | 2.3×10^-7^ | 81015 |
|  | 3 | 723 | 5.5×10^-7^ | 35261 |
|  | 4 | 1346 | 3×10^-7^ | 63259 |
|  | 5 | 1547 | 4.2×10^-4^ | 973 |
|  | 6 | 1518 | 2.4×10^-7^ | 57861 |
| 6 |  | 289 | 2.8×10^-3^ | 204 |
| 7 |  | 502 | 6.5×10^-7^ | 17654 |
| 8 |  | 30407 | 2.9×10^-6^ | 10314 |
